# Sequestration of macroalgal carbon: the elephant in the Blue Carbon room

**DOI:** 10.1098/rsbl.2018.0236

**Published:** 2018-06-20

**Authors:** Dorte Krause-Jensen, Paul Lavery, Oscar Serrano, Núria Marbà, Pere Masque, Carlos M. Duarte

**Affiliations:** 1 Department of Bioscience, Aarhus University, Vejlsøvej 25, DK-8600 Silkeborg, Denmark; 2 Arctic Research Centre, Aarhus University, Ny Munkegade 114, DK-8000 Århus C, Denmark; 3 School of Science, Centre for Marine Ecosystems Research, Edith Cowan University, Joondalup, Western Australia, Australia; 4 Global Change Research Group, IMEDEA (CSIC-UIB), Esporles (Illes Balears), Spain; 5 Departament de Física & Institut de Ciència i Tecnologia Ambientals, Universitat Autònoma de Barcelona, 08193 Bellaterra, Spain; 6 Oceans Institute & School of Physics, The University of Western Australia, Crawley, Western Australia 6009, Australia; 7 King Abdullah University of Science and Technology (KAUST), Red Sea Research Center (RSRC), Thuwal, 23955-6900, Kingdom of Saudi Arabia

**Keywords:** macroalgae, seaweed, Blue Carbon, carbon sequestration, export, connectivity

## Abstract

Macroalgae form the most extensive and productive benthic marine vegetated habitats globally but their inclusion in Blue Carbon (BC) strategies remains controversial. We review the arguments offered to reject or include macroalgae in the BC framework, and identify the challenges that have precluded macroalgae from being incorporated so far. Evidence that macroalgae support significant carbon burial is compelling. The carbon they supply to sediment stocks in angiosperm BC habitats is already included in current assessments, so that macroalgae are de facto recognized as important donors of BC. The key challenges are (i) documenting macroalgal carbon sequestered beyond BC habitat, (ii) tracing it back to source habitats, and (iii) showing that management actions at the habitat lead to increased sequestration at the sink site. These challenges apply equally to carbon exported from BC coastal habitats. Because of the large carbon sink they support, incorporation of macroalgae into BC accounting and actions is an imperative. This requires a paradigm shift in accounting procedures as well as developing methods to enable the capacity to trace carbon from donor to sink habitats in the ocean.

## 1. Introduction

Recognition of the role of vegetated coastal ecosystems as sites of intense carbon (C) sequestration and storage [1,2] led to the development of Blue Carbon (BC) strategies to mitigate and adapt to climate change through the conservation and restoration of these ecosystems [3–5]. Assessments of C sequestration and stocks in support of BC strategies have, thus far, been restricted to angiosperm-dominated ecosystems (saltmarshes, seagrasses and mangroves), which accrete sediments where C is stored [4]. Yet, a potentially large contribution of macroalgae to C sequestration was recently proposed [6]. Indeed, macroalgae form the most extensive and productive vegetated coastal habitats in the global coastal ocean, estimated to cover about 3.4 million km^2^ and support a global net primary production of about 1.5 Pg C yr^−1^ [6].

Because the large contribution of macroalgae to global ocean C fluxes was first pointed out more than 35 years ago [1], the neglect of macroalgae in current BC assessments identifies macroalgal C as the *elephant* in the BC framework. Here we review the role of macroalgae in the BC context. We do so by first summarizing and analysing existing evidence for this role and the stated reasons why macroalgal C is not included. We then evaluate whether macroalgal C fulfils the criteria that have rendered BC a successful strategy to mitigate and adapt to climate change.

## 2. Carbon sequestration by macroalgae

BC assessments have so far been focused on C sequestration within the habitat. While most macroalgae grow on rocky shores where sediment accretion does not occur, a significant fraction of macroalgal production is exported [7,8], to eventually reach shelf sediments, including those in angiosperm-dominated habitats [9–11], and the deep ocean, where it can be stored over significant time scales [6]. Hence, macroalgae do contribute to C sequestration, but this largely occurs in depositional areas beyond their habitats [6,12].

Macroalgae contribute substantially to the estimated organic C export to the open ocean of 2.4 Pg C yr^−1^ [8] with a first-order global estimate of 173 Tg C yr^−1^ of macroalgal C potentially sequestered in sediments and deep-sea waters, about 11% of macroalgal net C production [6]. This is comparable to the C sequestered by all other BC habitats combined [4] and a potential C sequestration of that magnitude cannot be ignored.

## 3. Macroalgae in the Blue Carbon literature

A search in Web of Science on 8 February 2018, using the search string ‘“Blue Carbon” AND (macroalga* OR seaweed*)’, retrieved 16 relevant publications (electronic supplementary material, table S1). The first paper was published in 2011 [13], five in 2017 (electronic supplementary material, table S1), and citations of the BC papers grew 18-fold (from 5 to 92 citations) between 2012 and 2017. This reveals an emerging interest in the topic, including the role of seaweed aquaculture and created seaweed habitats as potential BC resources (electronic supplementary material, tables S1 and S2). This search, however, did not capture papers published before the term ‘Blue Carbon’ was first introduced [3], such as the seminal 1981 paper by Smith [1], and recent studies discussing the role of macroalgae (electronic supplementary material, table S2).

The multiple mechanisms by which kelps and other macroalgae may promote C sequestration were already outlined in 1981 [1] (electronic supplementary material, table S2). Later studies pointing at a major role for vegetated ecosystems in C sequestration [2], which seeded the BC concept [3], acknowledged that estimates based on angiosperm-dominated habitats alone are conservative, as many macroalgae thrive on sandy and muddy seafloors where C may be buried (electronic supplementary material, table S2). The BC concept report also pointed at opportunities to contribute to mitigating climate change through macroalgal farming [3] (electronic supplementary material, table S2), while similar opportunities apply to wild harvest of macroalgae [14]. The role of macroalgal farming as ‘created BC habitat’ that may contribute to climate change mitigation and adaptation (electronic supplementary material, table S2) highlights that macroalgae are a component of BC that requires specific management [4] to deliver its potential as a C donor [12,15] (electronic supplementary material, table S2). However, in a recent study, Howard *et al*. [16] concluded that macroalgae cannot be ‘considered as part of a viable climate mitigation strategy’, a conclusion that was immediately challenged by Smale *et al*. [17] and is inconsistent with arguments in many papers (electronic supplementary material, table S2).

## 4. Many shades of Blue Carbon

An added difficulty to integrating macroalgae in BC science and policy is their broad phylogenetic and ecological diversity compared with the relatively uniform nature of the foundation plant species forming saltmarsh, seagrass and mangrove ecosystems. Macroalgae are a polyphyletic, operational category of organisms comprising four phyla (Rhodophyta, Phaeophyta, Chlorophyta and Cyanophyta) and about 60 orders (13 Phaeophyta, up to 30 Rhodophyta, 15 Chlorophyta and three Cyanophyta) [18–22] distributed in different kingdoms. Red and green algae (in some systematics) are categorized within the plant kingdom, brown algae within the Chromista kingdom, and blue–green algae in the bacteria kingdom. Macroalgae are, therefore, as different in evolutionary origin as elephants and *Boletus* mushrooms.

This evolutionary diversity translates into a huge diversity in forms and size, which carries functional consequences [23–25] affecting the fate of macroalgal C. Large, long-lived macroalgae, K-selected species [26] such as kelps and Fucales, have thick, leathery thalli with high ratio of structural to photosynthetic tissue and low nutrient content, and are more resistant to grazing and decomposition than r-selected, opportunistic species such as the sea lettuce, *Ulva lactuca* [23–25]. The slower turnover of the organic matter makes the large, long-lived macroalgae more likely to contribute to C sequestration [27]. Indeed, for kelp communities, on average 82% of the local primary production is exported to adjacent communities [28], compared with 43% of overall macroalgal primary production exported [7]. Red algae, such as crustose coralline algae, also K-selected algae, exhibit large resistance to grazing [23,24] and have a potential for long-term C storage [15,29] (electronic supplementary material, table S2). However, C sink estimates for these algae must account for the balance between the CO_2_ sequestered and CO_2_ emitted during calcification [30]. A recent comparison of marine macrophytes for their likely contributions to BC sequestration highlighted that macroalgae contain refractory compounds supporting long-term C storage but exhibit a much larger variation in tissue stability among macroalgal taxa relative to vascular plants, consistent with the larger diversity of cell wall structure and composition [15].

The thickness of macroalgal thalli is a predictor of their photosynthetic performance [31], growth rates [32], nutrient stoichiometry and decomposition rates [33], affecting their production and the lability of the C they produce and constraining their distribution relative to light availability. Remarkably, red coralline algae exhibit lower light requirements and, hence, typically grow deeper than other macroalgae [34] and are, therefore, likely to contribute most to global macroalgal extent, production [29,34], and possibly C export. Still, their net C sequestration must consider CO_2_ emission via calcification in addition to CO_2_ sequestration via organic C burial.

Whereas extent, production and recalcitrance are fundamental traits constraining the potential for sequestration of macroalgal C, realizing this potential depends on the likelihood that the C reaches environments suitable for C preservation as mediated by physical, biogeochemical and biological processes [35]. The intrinsic property that affects the physical dispersal of macroalgae is the ability for export and long-distance transport, which also varies among groups of macroalgae. While most macroalgae are negatively buoyant, many of the large brown algae, including Fucales and Laminariales, have buoyancy mechanisms such as pneumatocysts allowing the thalli to float. The coincidence of traits facilitates the long-range export of detached, relatively recalcitrant macroalgal C of K-selected species, which, after the degradation of the pneumatocysts, sink to the sediments or the deep sea where the C can be sequestered [6]. In contrast, r-selected opportunistic algae may decompose at faster rates and thus potentially support the supply of detritus to habitats comparatively less distant from the release site than their K-selected counterparts.

## 5. Do macroalgae meet the criteria for Blue Carbon?

Whereas the literature search described above clearly shows that macroalgal C contributes to C sequestration, and that, if properly managed, created macroalgal habitats can also contribute to climate change mitigation and adaptation [13,14,36], this has not sufficed to integrate macroalgae into BC initiatives. Exceptions are PR China, which has included seaweed aquaculture as created BC habitat in their recently launched national BC programme [37], and Korea, with a BC programme built around constructed seaweed habitat [13,38]. Yet, there is considerable disagreement as to whether macroalgae meet the criteria to be considered within the BC framework [16,17]. This disagreement is, to an extent, paradoxical, as C contributed by macroalgae is certainly included, not excluded, in assessments of C stocks in sediments of seagrass meadows, mangroves and saltmarshes. For instance, assessments using stable isotopes showed that 50% of seagrass sediment C is contributed by other primary producers, including macroalgae [9,11]. Likewise, seaweed has been reported to contribute up to 60% of C to Red Sea mangrove sediments [10]. There is no disagreement, to the best of our knowledge, that these contributions are indeed part of the BC potential of these habitats, which therefore identifies seaweed as donors of BC. Hence, the question is not whether or not macroalgal C is a BC resource, but how to include macroalgae in C accounting and BC schemes. The disagreement may be narrowed down to the identification of the donor sites of macroalgal C and the sink locations where macroalgal C accumulates and persists over relevant time scales.

### (a) Criteria for role of macroalgae in climate change mitigation actions

The first consideration is that the BC resource needs to be extensive and with a sufficiently high sequestration rate at a national scale. Kelps are extensively distributed throughout the temperate zone while large brown macroalgae (e.g. *Turbinaria* spp. and *Sargassum* spp.) abound along most tropical coasts. Estimates of the global extent of kelp forests range between 20 × 10^3^ and 400 × 10^3^ km^2^ [39], which represents only about 10% of the likely global area occupied by macroalgae [6,40]. While there is ample evidence that macroalgal C is sequestered in oceanic sinks beyond the macroalgal habitat [6], direct estimates of macroalgal C burial rate are not yet available.

A second requirement is that the BC resource must be ‘actionable’, that is, human action can drive a change in the amount of C being sequestered. Over the last 50 years, kelp forests have experienced a relatively small global decline of 0.018 yr^−1^ attributed to harvesting, pollution, invasive species and/or warming [41]. This raises the possibility that local management actions could avoid or revert part of these losses, thereby enhancing C sequestration. These actions include reducing eutrophication and other activities that hamper underwater light penetration, managing harvest of wild kelp stocks, and limiting bottom trawling, all of which are pressures leading to kelp decline [17,42]. Conserving and restoring macroalgal contributions to C sequestration also require actions at sink sites receiving the sequestered C, when these are at risk of being disturbed. Macroalgal farms represent an interesting option since their effectiveness depends entirely on the fate of the farmed production, which is entirely controlled; if that production is allowed to be exported and is subsequently sequestered then it falls under the same considerations as wild macroalgal production. PR China currently has over 1250 km^2^ of macroalgal farms, emphasizing the enormous potential for enhanced production and subsequent sequestration [14]. Moreover, macroalgal aquaculture can also reduce harvesting pressure on wild macroalgal stocks, thereby resulting in avoided emissions.

## 6. Old schemes for new carbon?

Current frameworks and regulations need to be reconsidered in order to include macroalgal C in mitigation and adaptation actions, as well as in national BC accounting. This would involve amending internationally agreed guidelines, such as the United Nations *2013 Supplement to the 2006 IPCC guidelines for national greenhouse gas inventories: wetlands* [43], to include macroalgae and guide stakeholders into articulating a role for macroalgae in national declared contributions.

To be included in greenhouse gas (GHG) accounting and mitigation programmes, macroalgae must meet the requirements set by the IPCC. Similarly, in GHG mitigation programmes (e.g. Australia's Emission Reduction Fund, http://www.cleanenergyregulator.gov.au/ERF), proponents obtain tradable credits for validated projects (e.g. re-forestation). Currently, the Verified Carbon Standard (VCS) is the most commonly used verification standard and includes a number of requirements for any project: the GHG emissions reduction or removal must be ‘real’, ‘measurable’, ‘permanent’, ‘unique’ and ‘additional’. Whether these schemes and their requirements are suited to dealing with macroalgae BC, as well as the angiosperm-dominated coastal ecosystems so far considered, remains to be assessed. Currently, the VCS specifically excludes allochthonous C stored within seagrass, saltmarsh and mangrove ecosystems from accounting.

### (a) Real and measurable

All GHG emission reductions and removals generated must be proven to have genuinely taken place. For macroalgae, this requires demonstrating enhanced sequestration at the sink site or reduced emissions at the donor site derived from action at the donor site (e.g. a kelp farm or conservation action). This is not unlike ‘off-site’ actions in other BC ecosystems, such as catchment management that results in avoided emissions in seagrass meadows by reducing eutrophication impacts. However, this requires the ability to identify macroalgal C in the ‘sink site’ and prove that the action led to the enhanced sequestration or reduced emissions. Yet, estimates of macroalgal C sequestration, including burial in ocean sediments, have, thus far, relied on indirect calculations, and their empirical verification requires the development of new methods. Specifically, emerging fingerprinting techniques, such as environmental DNA, open opportunities to trace macroalgal C burial beyond their habitats [44,45].

In addition, the way project boundaries are defined needs to allow for the separation of donor and sink sites characterizing most macroalgal BC. For macroalgal BC, to be accountable, requires certainty of the origin of the C, its sequestration in an area that is owned by the relevant jurisdiction, and the ability to claim allochthonous C. Ownership mechanisms are straightforward if the ‘sink site’ is within a country's exclusive economic zone (EEZ). However, if the sink site is beyond the EEZ, ownership of the sequestered C would need to be resolved. This may be relatively straightforward for nations with large contiguous EEZs (e.g. Australia, Chile, Argentina) or for relatively isolated island states, but more problematic for countries with small, contiguous EEZs sharing sink areas, such as those in semi-enclosed seas with reduced and adjacent EEZs (e.g. the Baltic and Mediterranean states). Apportioning the macroalgal C sequestered in these sink environments to one of the states' EEZ will be challenging or impossible, but sharing schemes, where multiple nations jointly claim the sequestered C, may be feasible.

### (b) Permanent

The GHG emission reductions or avoidance generated by actions need be maintained over time scales of 10–100 years [46]. Whereas macroalgal C is often considered relatively labile and, therefore, less suited to preservation than other types of BC, some macroalgal C can be preserved for up to millions of years as oil, as documented by the presence of Rhodophyta in oil shales [47], and over centuries to millennia in seagrass sediments [48,49]. Moreover, all macroalgal C reaching the deep sea (deeper than 1000 m) will meet the requirement of permanence, as defined above, regardless of the fate of the C, buried in sediments, grazed or mineralized or suspended in nepheloid layers, since this C will require centuries to return to atmospheric exchange [6].

### (c) Additional

GHG emission reductions and removals must be additional to what would have happened if the project had not been carried out. The procedures for demonstrating additionality are not, conceptually, different for macroalgae than for other BC habitats, except for the challenge of demonstrating the additionality of C emission reduction/sequestration at a sink site when the action was undertaken at a different, donor site.

### (d) Unique

Any credited C emission reduction must be unique and associated with a single GHG emission reduction or removal activity. Consequently, current schemes only credit autochthonous C, as there is a risk that allochthonous C may have been previously credited. This is problematic for BC ecosystems owing to the high degree of connectivity in marine environments, resulting in large inputs of allochthonous C [17]. For example, as discussed above, 50% of C in seagrass sediments is typically non-seagrass C [9]. This issue becomes even more significant for macroalgae, which primarily support C sequestration beyond the habitat where the conservation or habitat creation takes place, i.e. by definition, allochthonous C. Yet, the risk of double accounting allochthonous C in BC habitats only applies to C derived from land, as current schemes only credit marine C in sediments of BC habitats, and this sequestration is not accounted elsewhere. By contrast, some of the C credited in forests on land may eventually be exported to the ocean and this may be the reason for the current reluctance to credit allochtonous C in BC habitats. Incorporating macroalgal BC into accounting and mitigation strategies may therefore require a paradigm shift [17] in the accounting procedures, and more precision in defining the risks of double counting than just considering all allochthonous C questionable. Further studies fingerprinting the C of BC habitats and documenting connectivity between habitats will support such developments. This paradigm shift should also be applied to BC sequestered beyond seagrass, mangrove and saltmarsh ecosystems.

## 7. Climate change adaptation benefits of macroalgal habitats

The corollary to the conservation and creation of macroalgal habitats is that they contribute to climate change adaptation, including adaptation to sea-level rise and increased storm surges through the capacity of macroalgae to reduce water flow [50] and physical disturbance [42], promote sedimentation [51] and provide refugia of increased pH to calcifiers vulnerable to ocean acidification [52]. These adaptation benefits are delivered together with a number of services to coastal populations, including food supply through the fisheries supported by macroalgal habitats, which can help build resilience to climate change impacts. As an underlining of the societal service of macroalgae, the ‘kelp highway’ hypothesis assigns an important role to kelp habitats along the North Pacific shorelines from Asia into America as a reliable source of food during the migration of humans along a narrow corridor of ice-free shoreline, eventually leading to the colonization of North America as the world warmed some 17 000 years ago [53].

Whereas the emphasis of BC projects has been on mitigation, as these actions may link to credits and financial mechanisms providing resources for conservation and restoration, the value of macroalgae in supporting climate change adaptation should not be overlooked. Indeed, more nations are resorting to BC habitats for adaptation actions than for mitigation within their National Declared Contributions [54,55].

## 8. Knowledge gaps and directions for future research

The preceding review clearly identifies macroalgae as producers of BC and fails to identify an absolute reason for rejecting macroalgae as potential subject of BC projects. However, a number of challenges need be addressed to fully embed macroalgae within the BC paradigm. These challenges, forming a road map for science and management/policy agendas (table 1), require confronting the challenge of tracing and understanding the large export flux, estimated at 2.4 Pg C yr^−1^ [8], including C exported from angiosperm-dominated BC habitats [56], from productive coastal habitats to the open ocean. The agenda is not just required to inform BC options, as current inability to account for the fate of such large C flux is a major outstanding flaw in the global C budget [8,40]. Given the evidence of a major role of macroalgae in C sequestration, addressing these research and management/policy agendas is of consequence for climate change mitigation and adaptation.

**Table 1.** Science and management/policy agendas needed for including macroalgae in the BC paradigm and in BC schemes.

*The science agenda:*
1. Development of reliable tools to fingerprint the contribution of macroalgae to oceanic C sink sites beyond the habitats.
2. Field evidence, derived with the tools above, of macroalgal burial rates and stocks in oceanic C sink sites beyond the habitats.
3. Improved estimates of the global area and production of macroalgae, resolved to the level of major functional groups.
4. Case studies providing evidence of effects of management practices, in terms of protection and enhancement of macroalgal area and production, for C sequestration beyond the habitat, to meet the additional requirement.
*The management/policy agenda:*
1. A certification system of the CO_2_ emissions avoided and/or of enhanced sequestration through protection and restoration of habitats and through seaweed farming.
2. Revising crediting schemes to incorporate macroalgal C sequestered beyond these habitats.
3. Establishing fair mechanisms apportioning macroalgal C sequestered in shared deep sinks among the participating nations.

## Supplementary Material

Table S1. List of references on macroalgae in the Blue Carbon context.

## Supplementary Material

Table S2. Citations on macroalgae in the Blue Carbon (BC) context extracted from literature sources.

## Data accessibility

The datasets supporting this article are provided in the manuscript and as electronic supplementary material
