# Supplementary material for: Sequestration of macroalgal carbon: the elephant in the Blue Carbon room
Source: Biol Lett. 2018 Jun 20;14(6):20180236. doi: 10.1098/rsbl.2018.0236 (PMC6030603; doi:10.1098/rsbl.2018.0236)
Supplement: Table S1. List of references on macroalgae in the Blue Carbon context. [file rsbl20180236supp1.docx]

**Supplementary material to Krause-Jensen et al 2018 "Sequestration of macroalgal carbon: The elephant in the Blue Carbon room"**

**Table S1. List of references on macroalgae in the Blue Carbon context.** The references were retrieved from a search in Web of Science of literature, using the search string ' "blue carbon" AND (macroalga* OR seaweed*)' (accession date Feb 8, 2018). References are provided in chronologcal order. References # 3,4,5 and 18 were subsequently identified to not address this topic.

| 1. Chung IK, Beardall J, Mehta S, Sahoo D, Stojkovic S. 2011 Using marine macroalgae for carbon sequestration: A critical appraisal. *J. Appl. Phycol.* **23**, 877–886. (doi:10.1007/s10811-010-9604-9)  2. Chung IK, Oak JH, Lee JA, Shin JA, Kim JG, Park K-S. 2012 adaptation against global warming : Korean Project Overview. *ICES J. Mar. Sci.* **68**, 66–74. (doi:10.1093/icesjms/fss206)  3. Russell BD, Connell SD, Uthicke S, Muehllehner N, Fabricius KE, Hall-Spencer JM. 2013 Future seagrass beds: Can increased productivity lead to increased carbon storage? *Mar. Pollut. Bull.* **73**, 463–469. (doi:10.1016/j.marpolbul.2013.01.031)  4. Gleason FH, van Ogtrop F, Lilje O, Larkum AWD. 2013 Ecological roles of zoosporic parasites in blue carbon ecosystems. *Fungal Ecol.* **6**, 319–327. (doi:10.1016/j.funeco.2013.06.002)  5. Bourque AS, Kenworthy WJ, Fourqurean JW. 2015 Impacts of physical disturbance on ecosystem structure in subtropical seagrass meadows. *Mar. Ecol. Prog. Ser.* **540**, 27–41. (doi:10.3354/meps11505)  6. Sondak CFA, Chung IK. 2015 Potential Blue Carbon from Coastal Ecosystems in the Republic of Korea. **50**, 1–8.  7. Hill R, Bellgrove A, Macreadie PI, Petrou K, Beardall J, Steven A, Ralph PJ. 2015 Can macroalgae contribute to blue carbon? An Australian perspective. *Limnol. Oceanogr.* **60**, 1689–1706. (doi:10.1002/lno.10128)  8. Pettit LR, Smart CW, Hart MB, Milazzo M, Hall-Spencer JM. 2015 Seaweed fails to prevent ocean acidification impact on foraminifera along a shallow-water CO _2_ gradient. *Ecol. Evol.* **5**, 1784–1793. (doi:10.1002/ece3.1475)  9. Trevathan-Tackett SM, Kelleway J, Macreadie PI, Beardall J, Ralph P, Bellgrove A. 2015 Comparison of marine macrophytes for their contributions to blue carbon sequestration. *Ecology* **96**, 3043–3057. (doi:10.1890/15-0149.1.sm)  10. Ahmed N, Glaser M. 2016 Can ‘Integrated Multi-Trophic Aquaculture (IMTA)’ adapt to climate change in coastal Bangladesh? *Ocean Coast. Manag.* **132**, 120–131. (doi:10.1016/J.OCECOAMAN.2016.08.017)  11. Greiner JT, Wilkinson GM, McGlathery KJ, Emery KA. 2016 Sources of sediment carbon sequestered in restored seagrass meadows. *Mar. Ecol. Prog. Ser.* **551**, 95–105. (doi:10.3354/meps11722)  12. Krause-Jensen D, Duarte CM. 2016 Substantial role of macroalgae in marine carbon sequestration. *Nat. Geosci.* **9**, 737–742. (doi:10.1038/ngeo2790)  13. Kakuta S, Takeuchi W, Prathep A. 2016 Seaweed and seagrass mapping in Thailand measured using Landsat 8 optical and textural image properties. In *Journal of Marine Science and Technology (Taiwan)*, pp. 1155–1160. (doi:10.6119/JMST-016-1026-4)  14. Smale DA, Burrows MT, Evans AJ, King N, Sayer MDJ, Yunnie ALE, Moore PJ. 2016 Linking environmental variables with regionalscale variability in ecological structure and standing stock of carbon within UK kelp forests. *Mar. Ecol. Prog. Ser.* **542**, 79–95. (doi:10.3354/meps11544)  15. Ahmed N, Bunting SW, Glaser M, Flaherty MS, Diana JS. 2017 Can greening of aquaculture sequester blue carbon? *Ambio* **46**, 468–477. (doi:10.1007/s13280-016-0849-7)  16. Chung IK, Sondak CFA, Beardall J. 2017 The future of seaweed aquaculture in a rapidly changing world. *Eur. J. Phycol.* **52**, 495–505. (doi:10.1080/09670262.2017.1359678)  17. Duarte CM. 2017 Reviews and syntheses: Hidden forests, the role of vegetated coastal habitats in the ocean carbon budget. *Biogeosciences* **14**, 301–310. (doi:10.5194/bg-14-301-2017)  18. Liu S, Jiang Z, Wu Y, Zhang J, Arbi I, Ye F, Huang X, Macreadie PI. 2017 Effects of nutrient load on microbial activities within a seagrass-dominated ecosystem: Implications of changes in seagrass blue carbon. *Mar. Pollut. Bull.* **117**, 214–221. (doi:10.1016/j.marpolbul.2017.01.056)  19. Sondak CFA *et al.* 2017 Carbon dioxide mitigation potential of seaweed aquaculture beds (SABs). In *Journal of Applied Phycology*, pp. 2363–2373. (doi:10.1007/s10811-016-1022-1)  20. Zhang YY *et al.* 2017 Carbon sequestration processes and mechanisms in coastal mariculture environments in China. *Sci. China Earth Sci.* **60**, 2097–2107. (doi:10.1007/s11430-017-9148-7)  21. Smith S V. 1981 Marine Macrophytes as a Global Carbon Sink. *Science (80-. ).* **211**, 838–840. (doi:10.1126/science.211.4484.838) |
| --- |
