# Supplementary material for: Sequestration of macroalgal carbon: the elephant in the Blue Carbon room
Source: Biol Lett. 2018 Jun 20;14(6):20180236. doi: 10.1098/rsbl.2018.0236 (PMC6030603; doi:10.1098/rsbl.2018.0236)
Supplement: Table S2. Citations on macroalgae in the Blue Carbon (BC) context extracted from literature sources. [file rsbl20180236supp2.docx]

**Supplementary material to Krause-Jensen et al 2018 "Sequestration of macroalgal carbon: The elephant in the Blue Carbon room"**

**Table S2. Citations on macroalgae in the Blue Carbon (BC) context extracted from literature sources.** References are listed as numbers (#) with details given below in chronological order. References marked with * originate from the search in Web of Science (Table S1) while additional relevant references on the theme are listed without *. Comments, marked in italics, are in some cases given to supplement the cited information.

| **#** | **Citation** |
| --- | --- |
| [1] | "Marine macrophyte organic carbon production, biomass storage, burial, oxidation, oxidation-induced CaCO_3_ dissolution, and metabolically accelerated gas exchange across the air-sea interface are alternative expressions for a single complex carbon sink. Although the components of this sink do exist, their quantitative significance on a global scale is not yet known" |
| [2] | "This report shows that macroalgae have great potential for biomass production and CO_2_ bioremediation." |
| [3] | "Whereas macroalgae often grow in rocky shores, where substantial sedimentation rates are unlikely, many of them can also thrive in sandy and muddy bottom where C may be buried. However, the possibility of C burial in macroalgal beds has not been studied and, therefore, cannot be included in this assessment" |
| [4] | "Most macroalgal beds (including kelp forests) do not bury carbon, as they grow on rocky substrates where burial is impossible" but recommends "Implement win-win mitigation strategies in the ocean-based sectors, including to Encourage sustainable, environmentally sound ocean based energy production, including algae and seaweed" |
| [5]* | "Harvesting and appropriate use of macroalgal primary production could play a significant role in C sequestration and amelioration of greenhouse gas emissions." |
| [6]* | "Unlike other BC sectors (mangroves, seagrasses, and salt marshes) that accumulate and retain large amounts of carbon in sediments, kelp forests and seaweed beds do not have such sedimentary substrata. ...Because sediment is absent from kelp forests and seaweed beds...it is unlikely that the benefit of these marine resources can be addressed through carbon markets and management strategies that are strictly based on long-term (centennial) sequestration. However, there is substantial potential to develop seaweed CDM methodologies by capturing carbon through algal photosynthesis and using the resulting biomass as a substitute for fossil hydrocarbons" |
| [7] | "Most macroalgal stands develop on hard, rocky substrates, and — despite their high productivity (Table 1) and capacity to trap suspended particles — do not develop significant carbon deposits... most macroalgal communities grow on hard substrates and do not contribute to carbon sequestration except for the biomass that may be exported to the deep sea... Yet macroalgae can play a role in mitigating climate change if either wild or aquaculture crops are used to derive biofuels...Hence there is a potential for wild and cultured macroalgae to help mitigate climate change, while generating significant additional benefits" |
| [8]* | "Unlike other coastal BC habitats such as seagrasses, saltmarshes and mangroves, they do not develop their own organic-rich sediments, but may instead act as a rich carbon source and make significant contributions in the form of detritus to sedimentary habitats by acting as a "carbon donor" to "receiver sites" where organic material accumulates... We conclude that macroalgal communities have the potential to make ecologically meaningful contributions toward global BC sequestration, as donors, but given that the fate of detached macroalgal biomass remains unclear, further research is needed to quantify this contribution." |
| [9]* | "Seaweed beds and kelp forests are also likely candidates for BC sequestration and storage. However, unlike other BC ecosystems, these communities lack soil substrates. Thus, they do not retain large amounts of carbon in their sediments even though they can act as carbon sinks by reducing DIC [..]. Those ecosystems play important roles in maintaining biodiversity, providing coastal protection, improving water quality, and sequestering some carbon. We believe that, although SABs are artificial ecosystems, their services are similar to those gained from natural beds simply because both types sustain life on earth. [6] have also demonstrated that, as CO_2_ sinks, seaweeds can sequester/convert carbon within their biomass throughout their entire life span." |
| [10]* | "Macroalgae, due to their high rates of production, fragmentation, and ability to be transported, would also appear to be able to make a significant contribution as C donors to blue C habit... This study shows that marine macroalgae do contain refractory compounds and thus may be more valuable to long-term carbon sequestration than we previously have considered." |
| [11] | " .. the global production of free-living algae/crustose coralline algae was 0.4/1.2 × 10^9^ t C yr^−1^ suggesting a total potential carbon sink of 1.6 × 10^9^ tonnes per year. Coralline algae therefore have production rates similar to mangroves, salt marshes and seagrasses representing an as yet unquantified but significant carbon store, however, further empirical investigations are needed to determine the dynamics and stability of that store."  *Comment: While the authors acknowledge carbonate production to release CO_2_, their carbon sink estimate is for total (organic and inorganic) carbon buried, without accounting for the balance between CO_2_ sequestered and emitted.* |
| [12]* | "Seaweeds in integrated multi-trophic aquaculture can act as BC sink (Chung et al., 2013)." |
| [13]* | "This study demonstrates that restored seagrass meadows are an important sink of ‘BC’ for the lagoon system as a whole, as 10 yr after restoration the meadows were accumulating carbon from both sea- grass and non-seagrass sources."  *Comment: Macroalgae contributed 3 to 4 % of the organic carbon sequestered by restored seagrass meadows.* |
| [14]* | "To contribute to the research related to coastal BC and marine biodiversity as well as conservation and sustainable management of natural resources in coastal regions, the spatial distribution of benthic cover derived from satellite images can be the most practical tool for monitoring seaweed and seagrass beds. This study aimed at mapping the latest distribution of seaweed and seagrass in Thailand using Landsat 8 images."  *Comment: This study explores methods to quantify macroalgae as BC habitat.* |
| [15]* | "A rough estimate suggests that macroalgae could sequester about 173 TgC yr^-1^.... About 90% of this sequestration occurs through export to the deep sea, and the rest through burial in coastal sediments... macroalgae should be considered both in carbon accounting reports and within BC conservation and restoration strategies to mitigate climate change."  *Comment: Provides the first rough estimate of macroalgal contribution to C-sequestration.* |
| [16]* | "Seaweed fails to prevent ocean acidification impact on foraminifera along a shallow-water CO_2_ gradient ... Although *Padina* did not prevent adverse effects of ocean acidification, high biomass stands of seagrass or seaweed farms might be more successful in protecting epiphytic foraminifera."  *Comment: Discusses the potential role of macroalgae in climate change adaptation.* |
| [17]* | "Recent work has shown that macroalgae can function as ‘carbon donors’, as they produce and export material that is later assimilated by ‘BC’ habitats as allochthonous organic matter." |
| [18]* | "Unlike BC ecosystems, seaweeds or marine macroalgal communities do not develop their own organic- rich sediments as they primarily grow on hard rocky substrates, and thus, do not directly contribute to carbon sequestration [7]. Nevertheless, macroalgae have the potential to contribute to global BC sequestration by acting as a carbon donor to receiver sites where organic materials accumulate (Hill et al. 2015). Under certain conditions, macroalgae can serve as an effective CO_2_ sink due to their capacity for photosynthetically driven CO_2_ assimilation [5,6]... Seaweed harvesting from integrated multi-trophic aquaculture may release BC." |
| [19]* | "If measured according to the algorithm of forestry carbon, the annual contribution to the reduction in atmospheric CO_2_ by macroalgae and shellfish mariculture activities in China was equivalent to that of the afforestation of more than 0.5 million hectares of trees [..]. [..] estimated that about 4.5 million tons of carbon could be fixed via cultivating economic algae in China each year. If the output of macroalgae mariculture is increasing by 5% annually, the annual carbon fixation by macroalgae mariculture can reach 0.93 million tons of C by 2020."  *Comment: Fig. 2 shows the pathways of carbon sequestration from seaweed aquaculture (harvest, POC burial in sediments, and DOC export to the deep sea)* |
| [20]* | "There is considerable potential for increased drawdown of CO_2_ by SABs, though its effectiveness in amelioration of atmospheric CO_2_ increase will depend on the fate of the resulting biomass." |
| [21]* | *Comment: This study updates estimate of macroalgal export and burial within habitat based on updated production estimate and discusses the role of macroalgae in carbon sequestration based on the estimates by* [22]*.* |
| [23] | "Seaweed aquaculture, the fastest-growing component of global food production, offers a slate of opportunities to mitigate, and adapt to climate change. Seaweed farms release carbon that maybe buried in sediments or exported to the deep sea, therefore acting as a CO_2_ sink. The crop can also be used, in total or in part, for biofuel production, with a potential CO_2_ mitigation capacity, in terms of avoided emissions from fossil fuels, of about 1,500 tons CO_2_ km^−2^ year^−1^. Seaweed aquaculture can also help reduce the emissions from agriculture, by improving soil quality substituting synthetic fertilizer and when included in cattle fed, lowering methane emissions from cattle. Seaweed aquaculture contributes to climate change adaptation by damping wave energy and protecting shorelines, and by elevating pH and supplying oxygen to the waters, thereby locally reducing the effects of ocean acidification and de-oxygenation." |
| [24] | "The fraction of kelp-based carbon that is ultimately sequestered through burial in ocean sediments is still poorly understood [..]. The short life span of individual kelp plants (~1 year) and their lack of long-term carbon storage mean that they cannot act as effective long-term carbon sinks (Figure 2b; [..]) and are not considered as part of a viable climate mitigation strategy. Although harvesting kelp for human use (e.g. in cosmetics, paper, biofuel) could benefit climate mitigation by providing more sustainable alternatives to using products derived from petroleum or other natural resources (http://bit.ly/1gOLIhG), to date research on this topic is incomplete and is not being implemented at a globally relevant scale... other marine ecosystems and ecosystem components (i.e. corals, kelp, and marine fauna) do not act as substantial and/or long-term carbon sinks."  *Comment: Table 1: Total Carbon Sequestration by Kelp is listed as "Not Applicable"*. |
| [25]* | "We conclude that [Seaweed aquaculture beds] SABs can effectively contribute to CO_2_ mitigation by becoming carbon donors to other ecosystems and converting the biomass into a range of bioenergy products from biogas to liquid and solid biofuels. This would represent a win-win strategy for coastal BC ecosystems with the mitigation and adaptation measures that SABs could provide. The fate of exudation and fragments of seaweeds as a carbon sink in the deep sea should be assessed." |
| [26] | "...effectively managing blue carbon requires an appreciation of the inherent connectivity between marine populations and habitats... We suggest that policy frameworks, and the science underpinning them, should focus not only on carbon sink habitats but also on carbon source habitats, which play critical roles in marine carbon cycling and natural carbon sequestration in the oceans... policy frameworks such as the United Nations Framework Convention on Climate Change (UNFCCC) should evolve to incorporate processes that promote natural carbon sequestration by, for instance, acknowledging the role of carbon donors in crediting and management. " |
| [27] | ".. management actions in these donor ecosystems [e.g. macroalgal forests] would need to be clearly distinguished from baseline conditions, and a great deal of science would be required to meet the burden of evidence it would take to incorporate these donor ecosystems into the framework. In reality, this burden of evidence would likely be too difficult or too costly an undertaking for many countries, which makes it difficult to include carbon from these donor ecosystems. Another hurdle for inclusion in climate mitigation policy is the need for the carbon management activity to fall within a country’s jurisdiction... " |

1. Smith S V. 1981 Marine Macrophytes as a Global Carbon Sink. *Science (80-. ).* **211**, 838–840. (doi:10.1126/science.211.4484.838)

2. Gao K, McKinley KR. 1994 Use of macroalgae biomass production and CO 2 remediation: a review. *J. Appl. Phycol.* **6**, 45–60.

3. Duarte CM, Middelburg JJ, Caraco N. 2005 Major role of marine vegetation on the oceanic carbon cycle. *Biogeosciences* **2**, 1–8. (doi:10.5194/bgd-1-659-2004)

4. Nellemann, C., Corcoran, E., Duarte, C. M., Valdés, L., De Young, C., Fonseca, L., & Grimsditch G. 2009 Blue Carbon. A Rapid Response Assessment. United Nations Environment Programme, GRID-Arendal.

5. Chung IK, Beardall J, Mehta S, Sahoo D, Stojkovic S. 2011 Using marine macroalgae for carbon sequestration: A critical appraisal. *J. Appl. Phycol.* **23**, 877–886. (doi:10.1007/s10811-010-9604-9)

6. Chung IK, Oak JH, Lee JA, Shin JA, Kim JG, Park K-S. 2012 adaptation against global warming : Korean Project Overview. *ICES J. Mar. Sci.* **68**, 66–74. (doi:10.1093/icesjms/fss206)

7. Duarte CM, Losada IJ, Hendriks IE, Mazarrasa I, Marbà N. 2013 The role of coastal plant communities for climate change mitigation and adaptation. *Nat. Clim. Chang.* **3**, 961–968. (doi:10.1038/nclimate1970)

8. Hill R, Bellgrove A, Macreadie PI, Petrou K, Beardall J, Steven A, Ralph PJ. 2015 Can macroalgae contribute to blue carbon? An Australian perspective. *Limnol. Oceanogr.* **60**, 1689–1706. (doi:10.1002/lno.10128)

9. Sondak CFA, Chung IK. 2015 Potential Blue Carbon from Coastal Ecosystems in the Republic of Korea. **50**, 1–8.

10. Trevathan-Tackett SM, Kelleway J, Macreadie PI, Beardall J, Ralph P, Bellgrove A. 2015 Comparison of marine macrophytes for their contributions to blue carbon sequestration. *Ecology* **96**, 3043–3057. (doi:10.1890/15-0149.1.sm)

11. van der Heijden LH, Kamenos NA. 2015 Reviews and syntheses: Calculating the global contribution of coralline algae to total carbon burial. *Biogeosciences* **12**, 6429–6441. (doi:10.5194/bg-12-6429-2015)

12. Ahmed N, Glaser M. 2016 Can ‘Integrated Multi-Trophic Aquaculture (IMTA)’ adapt to climate change in coastal Bangladesh? *Ocean Coast. Manag.* **132**, 120–131. (doi:10.1016/J.OCECOAMAN.2016.08.017)

13. Greiner JT, Wilkinson GM, McGlathery KJ, Emery KA. 2016 Sources of sediment carbon sequestered in restored seagrass meadows. *Mar. Ecol. Prog. Ser.* **551**, 95–105. (doi:10.3354/meps11722)

14. Kakuta S, Takeuchi W, Prathep A. 2016 Seaweed and seagrass mapping in Thailand measured using Landsat 8 optical and textural image properties. In *Journal of Marine Science and Technology (Taiwan)*, pp. 1155–1160. (doi:10.6119/JMST-016-1026-4)

15. Krause-Jensen D, Duarte CM. 2016 Substantial role of macroalgae in marine carbon sequestration. *Nat. Geosci.* **9**, 737–742. (doi:10.1038/ngeo2790)

16. Pettit LR, Smart CW, Hart MB, Milazzo M, Hall-Spencer JM. 2015 Seaweed fails to prevent ocean acidification impact on foraminifera along a shallow-water CO _2_ gradient. *Ecol. Evol.* **5**, 1784–1793. (doi:10.1002/ece3.1475)

17. Smale DA, Burrows MT, Evans AJ, King N, Sayer MDJ, Yunnie ALE, Moore PJ. 2016 Linking environmental variables with regionalscale variability in ecological structure and standing stock of carbon within UK kelp forests. *Mar. Ecol. Prog. Ser.* **542**, 79–95. (doi:10.3354/meps11544)

18. Ahmed N, Bunting SW, Glaser M, Flaherty MS, Diana JS. 2017 Can greening of aquaculture sequester blue carbon? *Ambio* **46**, 468–477. (doi:10.1007/s13280-016-0849-7)

19. Zhang YY *et al.* 2017 Carbon sequestration processes and mechanisms in coastal mariculture environments in China. *Sci. China Earth Sci.* **60**, 2097–2107. (doi:10.1007/s11430-017-9148-7)

20. Chung IK, Sondak CFA, Beardall J. 2017 The future of seaweed aquaculture in a rapidly changing world. *Eur. J. Phycol.* **52**, 495–505. (doi:10.1080/09670262.2017.1359678)

21. Duarte CM. 2017 Reviews and syntheses: Hidden forests, the role of vegetated coastal habitats in the ocean carbon budget. *Biogeosciences* **14**, 301–310. (doi:10.5194/bg-14-301-2017)

22. Krause-Jensen D, Duarte CM. 2016 Substantial role of macroalgae in marine carbon sequestration. *Nat. Geosci.* **9**, 737–742. (doi:10.1038/ngeo2790)

23. Duarte CM, Wu J, Xiao X, Bruhn A, Krause-Jensen D. 2017 Can Seaweed Farming Play a Role in Climate Change Mitigation and Adaptation? *Front. Mar. Sci.* **4**. (doi:10.3389/fmars.2017.00100)

24. Howard J, Sutton-Grier A, Herr D, Kleypas J, Landis E, Mcleod E, Pidgeon E, Simpson S. 2017 Clarifying the role of coastal and marine systems in climate mitigation. *Front. Ecol. Environ.* **15**, 42–50. (doi:10.1002/fee.1451)

25. Sondak CFA *et al.* 2017 Carbon dioxide mitigation potential of seaweed aquaculture beds (SABs). In *Journal of Applied Phycology*, pp. 2363–2373. (doi:10.1007/s10811-016-1022-1)

26. Smale DA, Moore PJ, Queiros AM, Higgs ND, Burrows MT. 2018 Appreciating interconnectivity between habitats is key to blue carbon management. *Front. Ecol. Environ.* **in press**, 2016–2018. (doi:10.1002/fee.1765)

27. Sutton-Grier A, Howard J. 2018 Coastal wetlands are the best marine carbon sink for climate mitigation. *Front. Ecol. Environ.* **16**, 73–74. (doi:10.1002/fee.1766)
